# Using Realist Evaluation to Understand Process Outcomes in a COVID-19-Impacted Yoga Intervention Trial: A Worked Example

**DOI:** 10.3390/ijerph18179065

**Published:** 2021-08-27

**Authors:** Abby Haynes, Heidi Gilchrist, Juliana S. Oliveira, Anne Tiedemann

**Affiliations:** 1 Institute for Musculoskeletal Health, The University of Sydney and Sydney Local Health District, Camperdown, NSW 2050, Australia; heidi.gilchrist@sydney.edu.au (H.G.); juliana.oliveira@sydney.edu.au (J.S.O.); anne.tiedemann@sydney.edu.au (A.T.); 2 Faculty of Medicine and Health, School of Public Health, The University of Sydney, Sydney, NSW 2006, Australia

**Keywords:** realist evaluation, methodology, healthy ageing, fall prevention, yoga, telehealth

## Abstract

Realist evaluation offers a valuable way to understand how interventions function and thus how they can be improved and locally adapted. Consequently, realist evaluation is increasingly conducted in parallel with intervention trials. It comprises a clear philosophical foundation and view of causality, pragmatic mixed data collection methods, and a theory-driven approach in which hypothesised program theories are tested and refined. However, detailed methods for data analysis are seldom well-described in realist studies and no clear method for analysing and presenting realist evaluation data has yet emerged. In this methodological paper we use the worked example of our realist process evaluation of the SAGE yoga trial to illustrate an applied process of data analysis and presentation of findings. We show how we drew on other realist studies for ideas, provide examples of six key tasks involved in conducting a realist process evaluation (including coding data and structuring results) and describe strategies that did not work and our rationale for rejecting them. This detailed account of the decisions and methods that worked for us is intended to provide a practical and informed point of departure for researchers conducting a realist evaluation.

## 1. Introduction

Realist evaluation is a theory-driven approach to evaluation that aims to explain why policies and programs work (or not), for whom, and under which circumstances [1,2]. The use of realist evaluation in health research is expanding both in terms of popularity and application [3], largely due to its utility in providing actionable ‘real world’ information about how interventions produce outcomes. Realist evaluation can add value by improving the clarity, depth, and transferability of findings; provide evaluators with pragmatic techniques for dealing with context and complexity; and give implementers helpful tools and lenses for critically appraising programs and generating knowledge, including for scale-up, translation and longer-term sustainability [1,4].

This methodological paper adds to the literature on analysis in realist evaluation by providing a worked example of a realist process evaluation conducted in parallel with a randomised controlled trial of yoga classes for older adults aimed at preventing falls. We provide a stepwise overview of a realist process evaluation for researchers who may have limited experience of qualitative methods and who are interested in realist evaluation but have yet to use it as a method.

### 1.1. Realist Evaluation

Realist evaluation seeks to identify causal patterns comprising three inter-related concepts: “context”, “mechanisms” and “outcomes” (see Table 1 for definitions) [5,6,7]. These linked concepts are the building blocks of tentative program theories that describe how an intervention functions in context, i.e., which mechanisms the intervention strategies are (or are not) activating, and how this is mediated by contextual factors to generate outcomes [8,9]. Theoretical understanding of how programs work can strengthen intervention design, adaptation and evaluation; hone local implementation processes; and improve how interventions are targeted [10,11].

Methodologically, realist evaluation is “agnostic”, taking a pragmatic approach to data collection that usually results in the use of mixed methods [12]. Quantitative methods may be more effective for identifying outcomes (including variation in outcomes) and certain aspects of context, while qualitative methods such as interviews or focus groups tend to be more appropriate for investigating mechanisms (which coalesce around human reasoning) and identifying unanticipated aspects of context and outcomes [7,13].

Realist evaluation is increasingly conducted in parallel with intervention trials. Its use of different lenses of inquiry (inherent in mixed method approaches) can reveal richer, more comprehensive insights into how interventions function [3,14,15]. Quantitative methods are more strongly associated with trials but qualitative research can add value by improving trial recruitment practices, fine-tuning intervention design, implementation processes and outcome measurement instruments; and by explaining how the intervention functioned in relation to various contextual factors, thereby facilitating the transferability of findings to other settings [16,17,18].

The Realist And Meta-narrative Evidence Syntheses: Evolving Standards (RAMESES) group provide a range of materials to support realist evaluation, including valuable book chapters and papers on realism’s philosophical underpinnings and data collection strategies (e.g., [6,19,20]). RAMESES also provide reporting standards for realist evaluation that ask researchers for a detailed description of how data were analysed [2,21] but they do not provide guidance as to how analyses should be conducted. Other researchers explore the key conceptual considerations in realist analysis of qualitative and mixed methods data [22,23,24,25], and issues of practical application are examined in a few studies which attend to the more granular detail of managing and coding data, and the myriad decisions this entails [8,26]. Some recent empirical papers have focused on the use of qualitative data analysis software in this process [27,28]. As yet, no clear method for analysing and presenting realist evaluation data has emerged, possibly because each study demands a tailored approach and evaluators are wary of slavish adherence to a *pro forma*. However, greater attention to the practical detail of conducting realist evaluation in published studies would support transparency and the applicability of this valuable approach [26].

**Table 1:** Glossary of realist terminology.

Term	Description
Intervention activities	Intervention activities offer resources (services, ideas, information, opportunities, constraints) with intended usages, but the manner in which people respond to these resources varies, depending on context. Thus an intervention itself does not work; rather, the recipients make it work depending on how they perceive and act in response to the resources it provides [29].
Context	Context is conceptualised as any condition that existed prior to the intervention [30]. This includes aspects of the local or wider environment (physical, economic, cultural) but also any relevant characteristics and circumstances of the people taking part in the intervention. Realists assume that interventions take place within complex, open social systems [6].
Mechanism	Program mechanisms are intangible forces usually comprising changes in participants’ cognitive or affective reasoning (conscious or unconscious), attitudes and choice-making or in their capacities (e.g. new skills, ideas, information, perspectives, or sources of support) [31]. Mechanisms are causal tendencies which are activated by intervention strategies, provided contextual factors are conducive [6].
Outcomes	Realist evaluation investigates how intervention activities produce outcomes [6]: intended and unintended. In a process evaluation, the focus is on process outcomes: proximal impacts which influence intervention outcomes. Desired process outcomes are those considered to be prerequisites for a successful intervention [32].
CMO and ICMO configurations	Realist evaluation links the elements described above in Context-Mechanism-Outcome configurations so that the causal connections between them are evident. Some program evaluators add intervention activities to this causal chain, resulting in Intervention activities-Context-Mechanisms-Outcome configurations (ICMOs) [8,33,34].

### 1.2. The Intervention: Successful AGEing (SAGE) Yoga Trial

This worked example of a realist evaluation centres on a current randomised controlled trial involving 560 community-dwelling adults aged 60+ years. Participants are randomised to either: (1) the Successful AGEing (SAGE) yoga exercise program in which they attend 40 weeks of twice-weekly yoga-based exercise classes designed to prevent falls or (2) a seated yoga relaxation program. The primary outcome is rate of falls in the 12 months post randomisation. Secondary outcomes include mental well-being, physical activity, health-related quality of life, balance self-confidence, physical function, pain, goal attainment, and sleep quality. An economic analysis is comparing the cost-effectiveness and cost-utility of the two yoga programs. 

The SAGE yoga exercise program was designed to be delivered in yoga studios but, following social restrictions in response to the COVID-19 pandemic, the four groups that started classes in a studio were moved online and delivered via Zoom. All but 3 of the 46 participants at that time continued to attend classes online, and all but four of those who remained completed the full program. 

## 2. Materials and Methods

### 2.1. Study Design

A realist process evaluation was conducted in parallel with the trial (Trial registration: ACTRN12619001183178), focusing on the SAGE yoga exercise program. Process evaluation seeks to shed light on why an intervention was or was not effective, and how it (and similar interventions) can be improved for better contextual fit. This is particularly useful for new innovative trials such as SAGE where causal mechanisms are not yet understood [11], and for trials delivered across multiple sites resulting in interventions potentially being delivered and received in different ways [35]. 

This process evaluation sought to understand how SAGE functioned as both a studio-based program and an online program, and to explain why it worked well for most participants. Given these explanatory aims, we chose a realist approach because of its utility for surfacing and testing program theories. Our two-part research question was: In the SAGE yoga trial, what is working, for whom, and under what circumstances? What aided the transition from studio-based to online yoga classes?

The conduct of this realist process evaluation centred on the following six tasks:

1. Identify outcomes of interest
2. Develop initial program theories
3. Collect data to test these theories
4. Code data to capture causal relationships
5. Engage with the literature
6. Present findings in ICMO configurations

**Figure 1:**
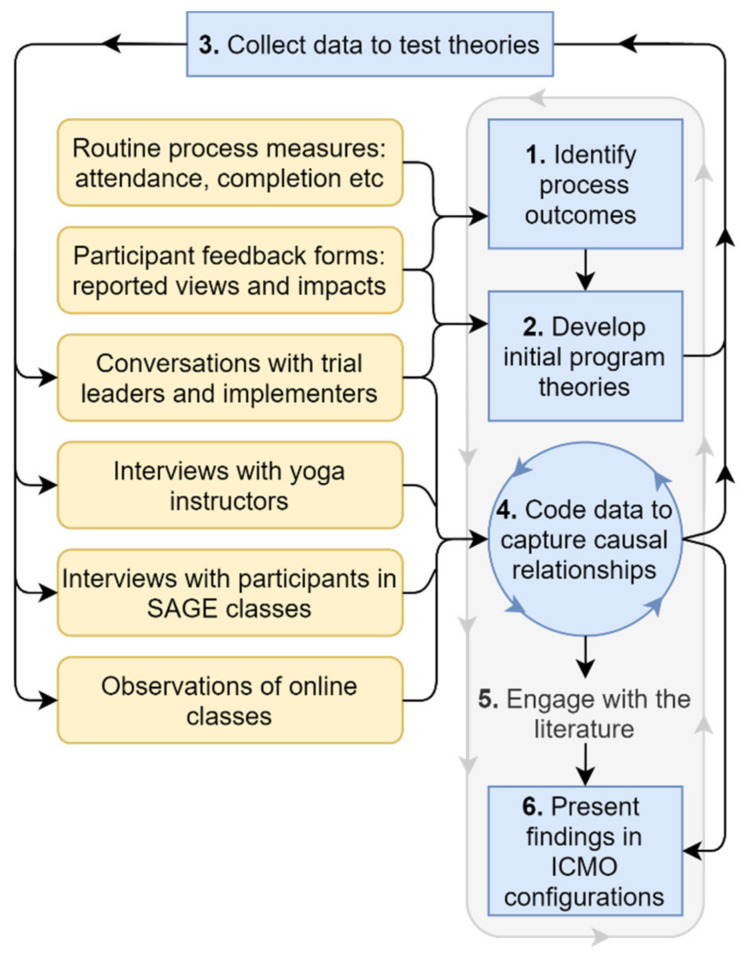
An overview of our realist process evaluation process.

We now explore each of these tasks in turn, using examples from our evaluation to illustrate how they were tackled. This process is illustrated in Figure 1.

#### 2.1.1. Task 1. Identify Outcomes of Interest

Realist evaluation usually starts by identifying outcomes of interest and seeks to explain how they were produced. Westhorp argues that in order to examine ‘what works for whom?’ it is necessary to define what constitutes ‘working’, i.e., program success [8]. The success of SAGE can be understood at two levels: process outcomes and formal trial outcomes. The trial was ongoing so the latter would not be available for many months; consequently, in this study we focus on process outcomes:

- Engagement with yoga program: attendance (including across transition to online classes), expressed commitment, motivation, and enjoyment.
- Self-reported health impacts: self-reported improved balance, flexibility, strength, mobility, stress reduction, sleep quality, sense of wellbeing, sense of achievement.
- Intention to continue yoga (or balance and strength-based physical activity).

Data sources for these outcomes included routine process measures which indicated high levels of program completion, and post-intervention participant feedback forms in which 87% of our targeted participants reported some degree of improved physical health or functioning, 87% reported improved mental health, and 78% reported improved balance. The qualitative data included comments about additional positive impacts such as improved flexibility, mobility, relaxation and/or sleep quality. Two further process outcomes were identified inductively as data collection progressed, and were refined with guidance from the literature:

d. Habit formation: routine practice of yoga as part of everyday life [36,37].
e. Physical literacy: motivation, confidence, physical competence, knowledge of and engagement in physical activity [38,39,40].

#### 2.1.2. Task 2. Develop Initial Program Theories 

As a form of theory-driven evaluation, the realist evaluation develops tentative initial program theories about how the program functions and uses these to guide data collection and analysis. We developed five initial theories (Table 2) derived from two sources:

- Discussion with the SAGE trial leaders and implementers about the underlying causal hypotheses that had informed the intervention design. These hypotheses derived from their research and professional expertise in the field of fall prevention, physical activity, and behaviour change, but had also evolved in response to information generated by the unfolding trial, including formal, and informal participant feedback and discussions with the yoga instructors.
- The literature on falls and older people’s engagement in yoga and yoga-based exercise, and physical activity more broadly. This provided ideas about possible program theories including likely mechanisms.

These rough working theories were intended to explain the three outcomes of interest we had identified at that point, and they were configured around intervention strategies and possible mechanisms (shown in italics). Punton et al. [4] recommend using rough program theories early on rather than developing detailed theories which may later prove to be irrelevant. Consequently, we waited until data collection was underway to flesh out contextual factors. We also wanted to retain the informal expression of these theories so we could ask interviewees about them conversationally.

Our hypothesis was that the mechanisms identified in these theories were activated in both studio-based classes and online classes, hence the high rates of program continuation despite the disruption and challenges of transition.

#### 2.1.3. Task 3. Collect Data to Test These Theories

##### Recruitment

We targeted participants who had been randomised to and enrolled in the four studio-based SAGE classes that later transitioned online and had thus experienced both types of classes. Sampling was intended to be purposive to support the realist approach to intra-group comparison in program evaluation [8], aiming for maximum coverage of all four groups, and maximum variation in engagement with the program within each group. However, of the 46 participants who completed the hybrid intervention, only 24 gave permission to be contacted for an interview and 3 of these did not respond to our invitation or declined due to illness. The study’s ethical approval did not permit us to approach people who had withdrawn from the trial. The three current yoga instructors were also invited to be interviewed.

##### Data Collection

Semi-structured interviews were conducted by phone with trial participants and two of the yoga instructors. The third instructor chose to be interviewed via video on Zoom. All interviews were conducted by a researcher with experience in qualitative research and realist evaluation (AH) who was not involved in the design or implementation of the SAGE trial.

Participant interviewees were encouraged to reflect on their experience of the hybrid program, including the differences between studio and online classes. They were asked to expand on scores and comments returned in their feedback form, and to explore their explanations for any intended or unintended impacts they identified [20]. Instructors were asked to comment on their experience of studio Vs online classes, how they had managed the transition and their perception of relative impacts. See Supplementary Files S1 and S2 for our final participant and instructor interview guides.

In accordance with the realist approach, we explicitly tested the program theories with participants [20]. In early interviews we introduced our five initial theories at the end of the interview by describing them in lay language and asking interviewees to critique each theory based on their experience of SAGE. Disagreement with or expansion of the theories was actively invited. For example, when discussing initial theory one (“*people stick with the program because they feel health benefits*”) we asked interviewees why they enrolled and stuck with the program in the early stages before any health benefits were felt. When discussing initial theory five (“*free classes are an incentive to give yoga a go and stick with it*”) we mentioned the anecdotal theory that people value services less when they are free [58]. If interviewees gave generalised responses, we prompted for concrete examples of their reasoning as these are more likely to reveal actual (rather than assumed) mechanisms [6]. 

In later interviews we adapted questions to take account of the evolution in our initial theories and to include emergent theories. In this phase we continued to prompt interviewees to critique the theories, often by highlighting variation in views from other participants. We also added a question about the home environment because it emerged as an important context for online classes.

Interventions-as-delivered often look quite different to interventions-as-theorised [59], and we were aware of the partial views of SAGE that we were obtaining from our interviewees, so one author (HG) conducted participant observations of online classes with different instructors. Screen shots were taken of one class by a non-participant observer and one instructor to show how participants and instructors viewed each other online. The document review included emails and texts from the three participants who withdrew during the transition period, and participants’ feedback forms. 

#### 2.1.4. Task 4. Code Data to Capture Causal Relationships

Realist data analysis is retroductive, that is, it seeks to explain outcomes by hypothesising causal pathways and testing these hypotheses to identify those that are most plausible [23,60]. In program evaluation this explanation comprises several elements, all of which must be identified in the analysis. These are: the intervention activities that trigger mechanisms, the mechanisms themselves (*how* the intervention works), the circumstances in which this occurs (context), and the effects produced by the interaction of those elements (outcomes). In addition to identifying those elements, data analysis must capture the relationship between them, i.e., the whole causal pathway. This presents challenges because the most common form of coding in qualitative analysis is *similarity* coding which forms categories of data that are like one another (similar views, experiences, or concepts) but fragments associations, processes, and causal relationships in the original structure of the data. Realist evaluation requires *contiguity* coding, which preserves these relationships [22,23].

Decisions must also be made about which elements of the causal pathway should be included. Traditionally, causal relationships in realist evaluation are expressed as Context-Mechanism-Outcome configurations (CMOs). In this approach the intervention activities are implicit because mechanisms are conceptualised as responses to resources provided by the intervention, thus a mechanism aggregates the intervention resource and people’s response to it. Dalkin et al. propose disaggregation, so that the intervention activity is less likely to be considered a mechanism in itself, and so the role of context as a mediating force between intervention resources and human reasoning is clearer [61]. One way of tackling this is to modify the CMO configuration to make the intervention activities explicit, for example, using Intervention activity-Context-Mechanism-Outcome configurations (ICMOs) [8,33]. We favoured this approach as the clearest depiction of program theory and because De Weger et al. [34] found that using ICMOs helped them focus on only coding causal relationships for which specific strategies had been implemented. This narrowed scope was appropriate for evaluating an intervention such as SAGE with well-defined intervention activities.

We reviewed and experimented with a range of approaches that would enable contiguity coding. Matrices have long been used in qualitative data analysis [62,63] and in several realist evaluations (e.g., [33,64,65]). For example, Westhorp [8] describes coding directly to her program theories and the ICMO configurations in each, and coding to align outcomes and mechanisms for each participant. This approach seems effective, as the outcomes are defined pre-data collection, and initial theories are well established so the evaluation is testing the extent to which they have explanatory power in the context of a specific intervention. We found that for our more exploratory study it was not flexible enough to support the rapidly evolving program theories, which required merging and adjusting some theories and generating (and rejecting) others. 

Bergeron et al. [27] coded transcripts in NVivo using discrete codes for context, mechanism, and outcome followed by sequences of matrix queries to connect CMOs, but they note that this process was cumbersome, demanding multiple matrices that did not link. Jackson and Kolla [30] also describe a complex staged analytical process, which they felt was best suited to a smaller dataset than ours. Other studies use separate CMO codes and then reconnect these into narratives [29,66,67]. We trialled this approach but felt we were losing the natural contiguity of the data. CMO coding also required that we made clear distinctions about what data belonged in each of these three categories. Similar to many others (e.g., [31,68,69]）, we found this hard to do with certainty, especially in the initial stages of coding, but we did find it helpful to follow Westhorp’s advice for identifying mechanisms:

*“any description of perceptions, beliefs, ‘logic in use’, emotional responses to situations, or understanding of a role that is relevant to the intervention may provide a clue”*

([8] p. 156).

**Figure 2:**
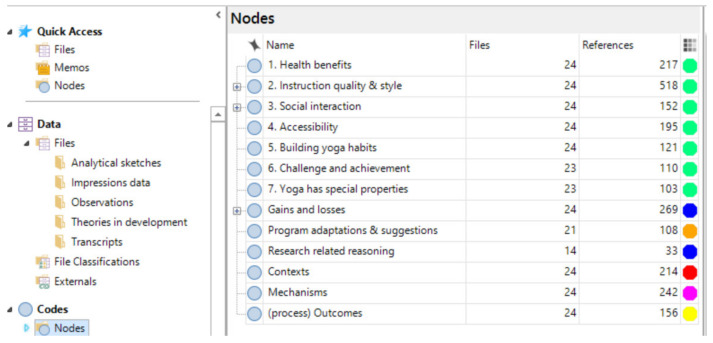
Final coding structure.

**Figure 3:**
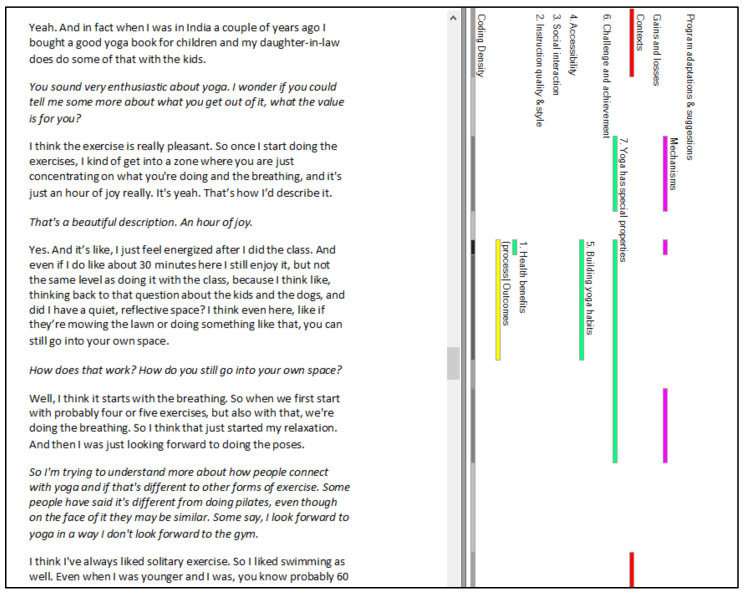
An example of coding to program theories + context, mechanism and outcome nodes in a participant transcript.

**Figure 4:**
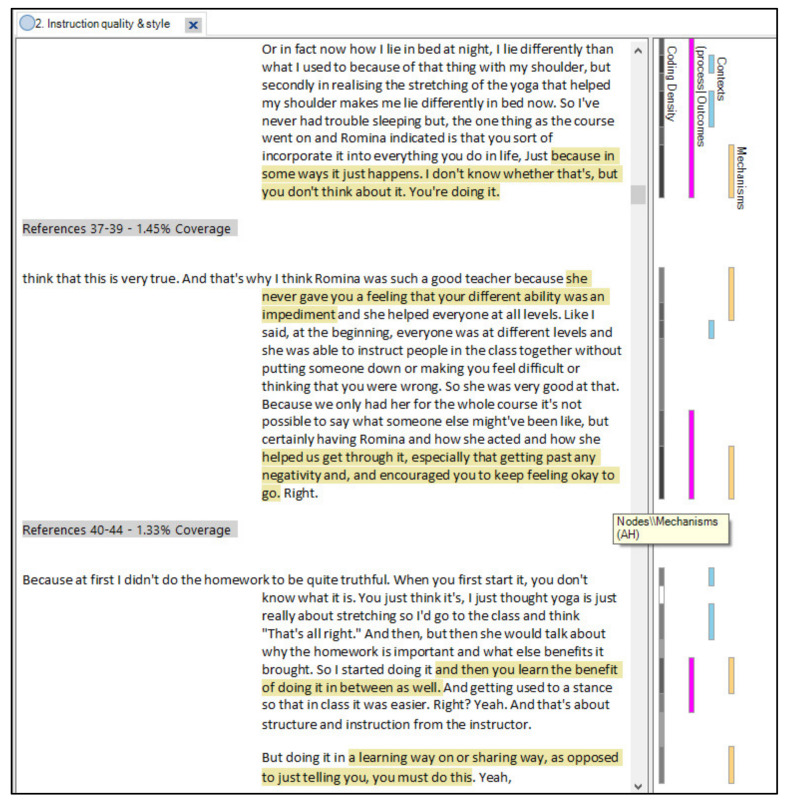
An example of how the natural connections between context, mechanism and outcomes can be preserved within the narrative text in a theory node. Here, the mechanism code is highlighted.

Consequently, we coded for program theories in NVivo using its traditional node function (one node for each theory), and supplemented this with additional nodes for context, mechanisms, and outcomes where likely candidates were identified (a similar approach was used by Gilmore et al. [26] and Dalkin et al. [28]). Figure 2 shows the final coding structure. This resulted in large chunks of relevant text being coded to each program theory which, when viewed in NVivo, allowed us to see Context, Mechanism, and Outcome codes as they were positioned within the original narrative account. Figure 3 shows how this looks within a participant transcript, while Figure 4 illustrates how CMO codes can be identified within text that has been coded to a program theory.

**Figure 5:**
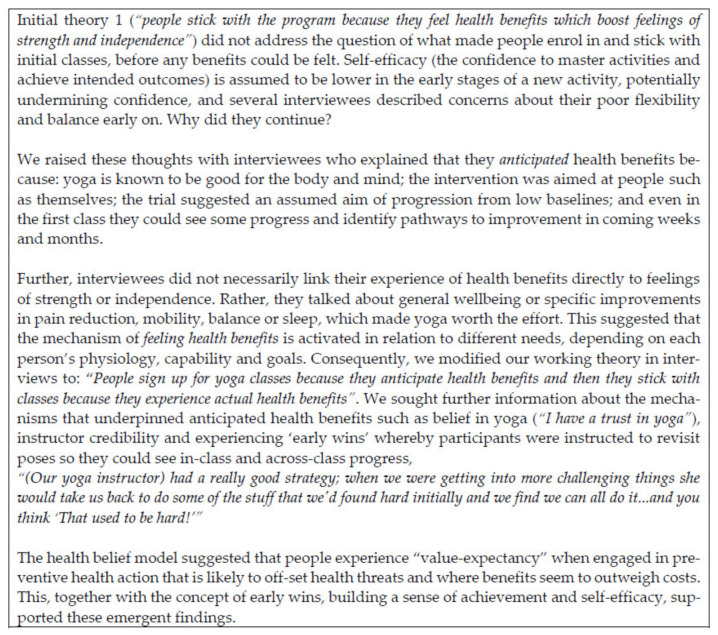
The Evolution of a Theory [70,71,72,73].

Two researchers (Abby Haynes and Heidi Gilchrist) both coded a proportion of transcripts independently using preliminary coding frames based on our five initial program theories and had frequent discussions about how each new transcript was reshaping the existing program theories and/or indicating others. See Figure 5 for an example. 

Given that the mode of yoga program delivery changed dramatically during the course of the intervention (transitioning from studio-based classes to online classes), we could not assume that the same mechanisms were activated throughout. Consequently, all data in which interviewees compared the two modes of delivery was double coded to a discrete node (Gains and Losses) as well as any relevant theory nodes. This allowed us to review all the comparative reasoning about what worked (or not) more efficiently. We erred on the side of caution in this task, coding to multiple theory nodes when we felt uncertain, and trying to identify likely contextual factors, mechanisms, and outcomes as we went (Figure 3). 

We kept a running analytic memo within NVivo to capture questions and ideas, and used annotations freely to link comments to a specific piece of text. Memos and annotations also served as a communication device between the two researchers conducting the analysis. 

#### 2.1.5. Task 5. Engage with the Literature

**Figure 6:**
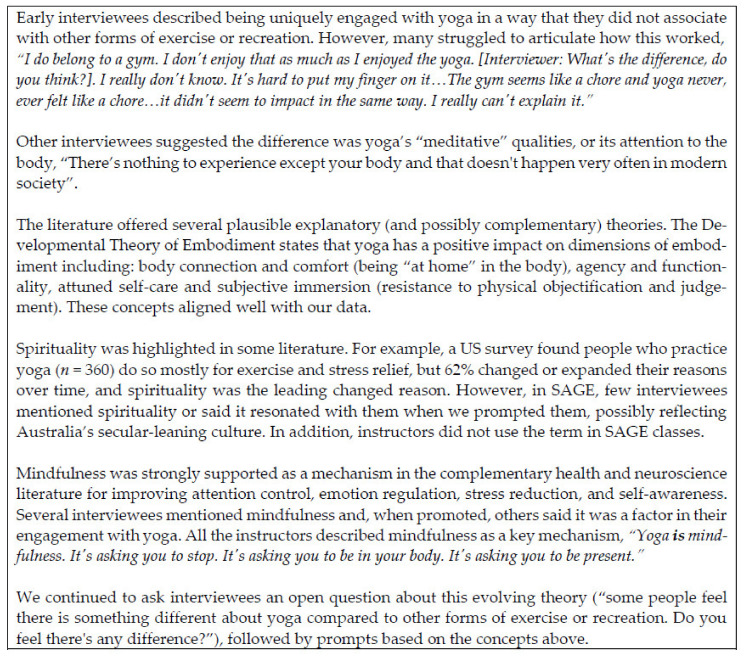
Using the Literature and Interviews to Develop a Theory [77,78,79,80,81,82,83,84,85].

**Figure 7:**
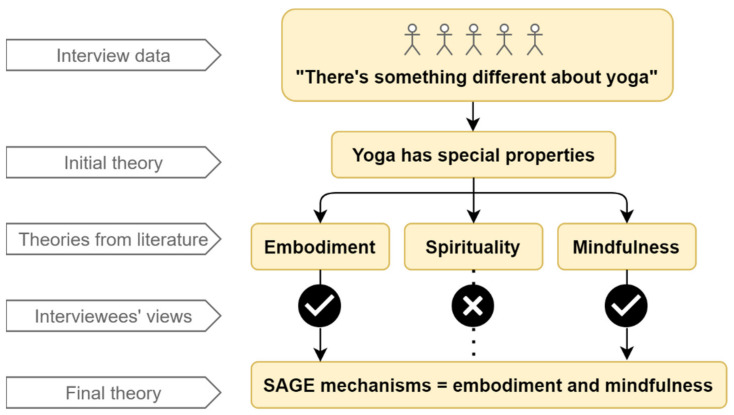
An overview of how we developed a program theory.

Initial program theories were stored in NVivo in a table with additional rows for other possible theories, columns for notes and queries and specific data sources, plus a column for revisions so that we could see how the theory was evolving. We investigated these initial theories and, later, our emergent theories in the literature in an effort to fine-tune them and to identify corroborating or disconfirming findings from other studies. This involved searching using likely keywords in Google Scholar and opportunistically following citations of theoretical or empirical studies that suggested plausible explanations for our findings. Others have conducted one-off replicable searches (e.g., [74,75,76]), but we chose an iterative exploratory search strategy where we followed ‘clues’ suggested by the program designers and, later, our interviewees throughout tasks 2, 3, and 4. This allowed us to review ideas from wide-ranging disciplines and unexpected sources (e.g., blogs) in relation to our initial and emergent theories. See Figure 6 for an example of a rough emergent theory that was honed initially via the literature and then tested with interviewees. Figure 7 provides an overview of this process.

Analysis was conducted in parallel with data collection. As interviews progressed and our theories evolved, we merged two initial theories, expanded one and added three additional theories to the final coding frame (Figure 2).

#### 2.1.6. Task 6. Present Findings in ICMO Configurations

After all transcripts were coded, we examined our theory codes to see if they would benefit from sub-coding (using *child nodes* in NVivo) but concluded that this would produce thematic categories rather than greater insights into the program theory suggested by that data. Consequently, we used memos to distil the contents of each theory node and to identify ICMO (Intervention activity-Context-Mechanism-Outcome) configurations. 

We moved between the data in each theory node and the separate C, M, and O nodes, our memos and annotations, and the literature synthesis in our theory development table, bearing in mind the possibility of any additional or alternative explanatory concepts that related to each theory. Two researchers (Abby Haynes and Heidi Gilchrist) worked on this independently and met to develop concordance. Observational data confirmed that the intervention strategies hypothesised to activate mechanisms were being delivered across different instructors’ classes, and this was corroborated by information from participants and instructors. We held periodic workshops with the wider research team to engage in critical dialogue about the emergent findings and to guard against “theory-induced blindness” in which data is overlooked because it does not fit initial hypotheses [8]. 

Lastly, we worked across the individual ICMO tables to develop a single table that distilled our entire data set, aiming to express these findings

*“…at a middle level of abstraction: specific enough to clearly explain the phenomenon, and general enough to apply across cases of the same type”*

([86] p. 3).

However, even after minimising overlapping concepts and repetition, the table was dense and occupied an entire page of A3 paper, making it hard to read easily and potentially overwhelming. This presentation also lacked coherence in relation to the overarching program theories that had formed the basis of our investigation. Work by Punton and colleagues [33] suggested an alternative approach in which ICMO configurations are attached to a “catchy” thematic title that encapsulates each program theory. This provides an entry point to the denser ICMO material and provides a clear structure for the more descriptive narrative account that follows the table. We found this meant we could be more succinct in the table itself, relying on the description for more nuance and exploration of complexity than the linear ICMO configuration format afforded [4]. We also used subheadings to make the table more accessible to readers unfamiliar with realist approaches.

### 2.2. Research Rigour

To strengthen the research rigour, we addressed the tasks in Ronkainen and Whiltshire’s framework for assessing validity in realist research in sports and exercise psychology [87] (Table 3). 

### 2.3. Ethical Approvals and Consent

Ethical approval for this study was provided by the University of Sydney Human Research Ethics Committee, reference 2019/604. Interviewees gave prospective informed consent to take part in an interview at the trial commencement and confirmed this in their post-trial feedback form. They also gave verbal consent to the trial administrators for their details to be passed on the interviewer and confirmed this consent in the interview itself after information about audio recording and use of deidentified data had been stated. 

This paper adheres to the reporting standards for realist evaluations developed by the Realist And Meta-narrative Evidence Syntheses: Evolving Standards (RAMESES) group [2]. 

## 3. Results

Twenty-four interviews were conducted: 21 with trial participants and 3 with yoga instructors. Interview duration ranged from 29 to 66 min with an average of 50 min. Fifteen trial participants were female (71%), reflecting the gender ratio in the trial, and aged between 61 and 80, with an average age of 68. This sample included four participants who stopped attending online classes, those who gave negative survey feedback and those with pronounced physical limitations such as osteoarthritis, scoliosis and Haglund’s deformity. The 3 yoga instructors were all female with over 15 years’ experience of delivering Iyengar yoga to groups, including those with older people. Analysis included data from 46 participant post-intervention feedback forms.

We identified 16 mechanisms. Contextual factors, especially people’s belief in the efficacy of yoga, their physical health, and domestic circumstances, played a powerful role in determining who the program worked for. The COVID-19 pandemic was a shared context that most likely enhanced engagement with online classes by limiting access to other forms of activity and connection. 

Table 4 provides an overview of our developing ICMO configurations. This analysis is ongoing, and it is likely that these theories will continue to evolve as we continue to move back and forth between our data and alternative theories in the literature to identify the most plausible and clearly articulated ICMO configurations. A further table will most likely be used to explore the comparative data between studio-based and online classes, and each program theory will be discussed narratively to explore the results in more detail. Consequently, the final results of the process evaluation, which will be presented in a subsequent paper, may be expressed differently.

## 4. Discussion

Our discussion focuses on four challenges in realist evaluation and how we tackled them in our realist process evaluation of the SAGE yoga trial.

### 4.1. Coding That Captures Relationships between Different Elements of the Program Theory

Realist evaluation requires an analytical approach that can simultaneously identify patterns of causal relationships and discrete elements within these relationships. The need to code for both contiguity and similarity presents analytic challenges. We described various strategies that have been used to tackle these challenges and present a detailed account of the methods that were identified as most fit-for-purpose for our study. Coding to whole program theories using nodes in qualitative data analysis software provided sufficient structure and flexibility to capture data related to evolving program theories. Others have also described how qualitative data management software can support and lend transparency to the *“messy”* and *“convoluted”* coding process by including both discrete constructs and narrative accounts [28].

### 4.2. Integrating Theory into the Ongoing Analysis

The use of theory presents another challenge. Realist evaluation employs an analytical process (retroduction) in which coding must be sensitive to emergence in the data but is also informed by working hypotheses and wider theories. This

*“involves constant shuttling between theory and empirical data, using both inductive and deductive reasoning”*

([88] p. 374).

The movement between inductive and deductive coding is common in some other research approaches where qualitative data is used, such as thematic analysis, content analysis and case study research; consequently, this aspect of analysis may be familiar to experienced qualitative researchers. However, the identification and critique of plausible theories throughout this process adds a layer of complexity. Despite considerable emphasis in the realist literature on the need to search for and iteratively test program theories against plausible rivals, very little guidance is offered about how to do this [89]. The one-off systematic procedures used in some studies seemed poorly suited to our exploratory approach in which we attempted to refine emergent theories (and our process outcomes) iteratively via further interviews and the literature. This pragmatic approach to following ‘clues’ in the interview data was more flexible but may have missed relevant studies and did not formally assess studies for quality. 

### 4.3. Identifying Clear Process Outcomes 

Much of the realist evaluation literature draws attention to the challenges of differentiating between intervention activities, context, mechanisms, and outcomes [90]. This is complicated in a process evaluation by the ‘interim’ focus of the evaluation on proximal outcomes that are considered to be causal mechanisms for generating the longer-term intervention outcomes [32]. We identified three process outcomes before the interviews started, and two further during interviews, but these were not clear cut. For example, should *improved health and wellbeing* be categorised as a mechanism or process outcome or summative outcome? We concluded that it plays a vital role in all three categories, but in different forms. Participants’ psychological *response* to anticipating and experiencing health benefits is a mechanism (which we called *value-expectancy* because health impacts were the key consideration in cost-benefit analyses about whether yoga was worth the effort). Given that improved health and wellbeing functions on a continuum, it is also a process outcome (because it contributes to motivation and adherence) and a final outcome indicating the success of the intervention along specific dimensions of health. Westhorp argues that

*“this is consistent with realist philosophy in which the same thing can be context, mechanism or outcome depending on the particular analysis being undertaken”*

([91] p. 378).

Thus, ICMOs are not self-evident and must be *“stitched”* together during analysis [33]. This involves understanding that elements in the configuration can *“slide”* depending on which point in the causal chain we are focusing our attention, thus an outcome or a context in a process evaluation may function as a mechanism when investigating longer-term outcomes [31,92].

### 4.4. Presenting Findings Accessibly

The purpose of realist evaluation (and synthesis) is to present clear causal explanations that articulate the relationships between context, mechanisms, and outcomes [2,31,34]. Methods for doing this vary widely in the literature, ranging from tables to flow charts and graphic depictions, many of which adapt the CMO configuration to include intervention activities more explicitly. We describe the difficulty we had in presenting a succinct and accessible overview of our findings due to the number of intervention activities, contextual factors, and mechanisms that our granular analysis produced. The associations between the elements in our ICMO configurations were also not always discrete: multiple aspects of the intervention worked synergistically to produce mechanisms. We found that attaching our configurations to a named program theory helped us to cluster ICMOs more efficiently, and focus on the aspects that were most relevant to our research questions. We were encouraged by Pawson’s advice that it is impossible to specify and investigate all the possible theories that inform the design and delivery of an intervention, thus evaluation must focus only on those that seem most likely to have profound causal implications, and which identify areas where modifications can be made [5]. He concludes that

*“the end result will be partial knowledge about partial improvements we can make in the delivery and targeting of social interventions – quite an achievement”*

([5] p. 112).

## 5. Conclusions

Realist evaluation—comprising a clear philosophical foundation and view of causality, purposeful mixed data collection methods and retroductive analytical strategies—offers a valuable way to better understand how interventions function and thus how they can be improved and locally adapted. Specific methods for analysing data are seldom well-described in realist studies, but moves are now afoot to address this gap and shine a light on applied analytical strategies. This paper adds to that movement. It draws on other realist studies for ideas, provides examples of key tasks involved in conducting a realist process evaluation, and describes strategies that did not work and our rationale for rejecting them. While this approach is unlikely to be directly transferable—research strategies must be tailored to each study [28]—this detailed account of the decisions and strategies that worked for us may provide a practical and informed point of departure for others.

## Figures and Tables

**Table 2:** Initial program theories about how SAGE works for most participants.

Initial Program Theory		Supporting Theories from the Literature
1.	People stick with the program because they *feel health benefits* (e.g., improved balance and mobility) which boost feelings of strength and independence	PA generates feelings of physical and psychological wellness [41], and yoga does this particularly effectively, including for older people [42,43]
2.	The *perceived quality of yoga instructors* is important for making people *feel safe and confident.* This includes instructors who understand the needs of older people	The quality of instructors affects older people’s feelings of safety and confidence in PA [44]. The concept of therapeutic alliance is key to understanding instructor/participant relationship quality [45]
3.	Tailoring of the classes is crucial. People must *feel they can participate according to their abilities and health needs*	Tailoring of PA programs is a key motivator in adherence [46,47]. Fall prevention interventions should be tailored for targeted recipients to maximise safety and effects [48]
4.	*Social connections* are an incentive and may add to the enjoyment of classes	Many empirical studies [47,49,50,51,52] and theories back this concept including self-determination theory [53] and the upward spiral theory of lifestyle change [54]
5.	Free classes are *an incentive* to give yoga a go and stick with it long-term	Subsidised costs increase participation in PA [55,56]. The ‘zero price effect’ [57] increases the perceptions of value

**Table 3:** Framework for validity in realist research in sports and exercise psychology.

Key Questions Re Validity Criteria	Tasks for Establishing Validity	Methods Used in This Study
How empirically adequate is the research account?	Establish descriptive validity	Transcription correction Timely note-taking re-emergent theories Multiple researchers engaged in coding and reviewing accuracy of findings against the data
	Guard against data collection limitations	Purposive sampling for maximum coverage Multiple methods triangulation Evolving interview questions taking account of new theories and concepts
How ontologically plausible is the research account?	Engage with theoretical explanations of the empirical evidence	Development of tentative initial theories Theory-checking with interviewees Continuous exploration of relevant existing theories in the literature
	Take account of context and complexity	In-depth exploration of context from multiple perspectives: diverse participants and intervention deliverers Particular attention to COVID-19 impacts
	Engage with competing alternative explanations of the evidence	Critical reflexivity Workshops with “critical friends” Inviting alternative theories from interviewees Multi-researcher coding Searching for alternative theories
How much practical utility does the research account have?	Findings are used to suggest practical real-world actions	Practical responses to the findings will be addressed in the main evaluation paper
	Those practical actions are likely to have a meaningful impact	The theoretical and empirical basis of the trial (which draws on demonstrably successful intervention strategies) plus self-reported benefits from participation and the scalability of the intervention indicate likely impact

**Table 4:** An overview of how the hybrid SAGE yoga program worked for current participants.

Program Theories	Intervention Activities What Did We Do?	Context Who Did It Work for?	Mechanisms How Did It Work?	Outcomes With What Process Effects?
It is worth the effort	Program of Iyengar yoga-based exercise with progressively challenging poses designed to prevent falls in older people	· The program attracted people who believed in the efficacy of yoga and who had interests in healthy ageing and/or tackling fall-related physical decline · It best suited those with physical capabilities in the moderate range who had manageable levels of pain · People who believed the Zoom interface impeded the effectiveness of yoga instruction valued online classes less than studio-based classes	· Value expectancy · Therapeutic alliance · Achievement/Mastery	· Engagement with SAGE: attendance (including across transition to online classes), expressed commitment and enjoyment · Self-reported improvements in balance, flexibility, strength, mobility, stress reduction, sense of wellbeing, and/or mindfulness · Habit formation: routine practice of yoga as part of everyday life · Physical literacy: physical competence and confidence (self-efficacy), including the creation of transferable skills, and motivation to engage in physical activity · Intention to continue yoga (or strength-based physical activity)
Impelling instruction	Experienced instructors deliver the program, individualising it for participants’ different capabilities			
Other people help	Group classes with a maximum of 18 people WhatsApp forum for each group when classes moved online	· Group classes worked for those who valued social interaction and/or shared experiences. This was enhanced by friendly group members of a similar age. · Studio-based classes suited those who liked to benchmark their physical competence and/or peer-audit their poses	· Shared experience · Social connection · Social comparison · Peer checking	
Putting yoga within reach	Free classes in local yoga studios... ... then online via Zoom with tech support from the SAGE team	· Those with easy access to a participating yoga studio · Online classes worked for those with suitable home environments and tech confidence or with hesitancy but openness to trying Zoom with support · COVID-19 restrictions generally seemed to enhance engagement with online classes	· Accessibility · Convenience · Gratitude	
Building yoga habits	Twice-weekly classes over 12 months with: · flexible ‘make up’ classes · program-specific homework tools and encouragement · goal-setting for mobility	· SAGE’s structure worked for people who prioritised and could commit to the schedule · Homework suited those keenest on progression and/or their instructor’s approval · Flexible classes were used by those with career commitments, travel plans, injury or illness · Goal-setting did not seem to work in SAGE	· Purposeful structure · Momentum · Accountability · Continuity	
Yoga has special properties	The SAGE program utilises core Iyengar yoga practices	· This worked best for those who were open to yoga as a holistic practice	· Embodiment · Mindfulness	

## Data Availability

The qualitative datasets generated during this study are not publicly available or available on request because they contain personal identifiable information about participants.

## Supplementary Materials

The following are available online at https://www.mdpi.com/article/10.3390/ijerph18179065/s1, Supplementary file S1. Interview guide for SAGE yoga trial participants, Supplementary file S2. Interview guide for SAGE yoga instructors.
