# Supplementary material for: Using Realist Evaluation to Understand Process Outcomes in a COVID-19-Impacted Yoga Intervention Trial: A Worked Example
Source: Int J Environ Res Public Health. 2021 Aug 27;18(17):9065. doi: 10.3390/ijerph18179065 (PMC8431647; doi:10.3390/ijerph18179065)
Supplement: Supplementary file 1 [file ijerph-18-09065-s001.zip › ijerph-1334644-suppl-xml3.pdf]

Participant Number: ______________

SAGE yoga trial INTERVIEW GUIDE

Thank you for agreeing to take part in this interview. As you know, we're evaluating the yoga program that you've been taking part in for the past 12 months. We hope that learning more about your experience will help us and other researchers design better programs like this in the future. Our conversation today is confidential which means that when we talk about or publish the results of this evaluation we won't use any information that reveals your identity, including with your yoga instructor. Does that sound OK?

Before we start, I want to let you that I'm **audio recording** our conversation so that I capture your views accurately. Is that OK? Thank you. ☐

Also, can I please double-check that you understand the purpose of this interview and you're **happy to take part**? Great – thanks! ☐

Part A. Reasons for participation

1. Thinking back to when you first heard about the yoga program, what made you decide to take part? What were you hoping to get out of it?

2. What were your views about yoga at the time?

3. What about falls – was that something you had been thinking about?

Part B. Experiences of participation

4. We're keen to learn as much as possible about what worked and what didn't work for you in the yoga program. I'll ask you some specific questions about that in a moment. But I wonder if you could start by telling me what your experience of the program was like overall.

5. As you know, due to COVID-19 the yoga classes were changed. You attended face-to-face classes then *took part in online classes/chose to do yoga on your own*. How would you compare the experience of doing yoga in those different ways? What aspects of each work for you? What doesn't work so well? You said on your feedback form that you preferred ____. Can you tell me more about that?
    a. [if applicable] How do you feel about not being able to attend face-to-face classes due to social restrictions?
    b. [if applicable] What about your connections with peers and the instructor from the group exercise class – has that been affected? Were you able to maintain social connections in the online classes? Do you think it helped that you'd already met face-to-face?

> Make sure questions 5 is considered for both face-to-face and online classes or self-directed practice: we want to understand the differences

Part C. Aspects of the yoga program

Now I'd like to ask you a few more questions about the detailed feedback you kindly gave us on your feedback form:

6. You scored the yoga instructor ___ out of 10 for how well they delivered the yoga program. Why was that? What was good about her? How could the instruction have worked better for you?

7. You rated the online yoga classes ______ out of 10. Can you expand on that? What was good about them? What could have made them work better for you?

8. When you were asked *'Do you have any other comments'* about the program, you said ____

____ Can you expand on that?

Part D. Impacts and maintenance

- mental well-being
- PA levels
- attitude to PA
- quality of life
- balance
- self-confidence
- physical function
- pain
- goal attainment
- sleep quality

9. Do you think anything has changed physically or mentally because of taking part in this yoga program?

10. On your feedback form you rated the program ___ out of 10 for how beneficial you found it. You said:

____
Can you tell me more?

11. What are your views about yoga now?

12. What about preventing falls - has that changed in any way? Is it an issue for you personally?

13. Thinking about the future now… What do you feel will happen with your yoga practice over the next 12 months? Do you see yourself keeping it up? What would help?

14. Now that you've done the yoga program, what do you think about the goals you set at the beginning? Would you set the same goals again, or would you aim for anything different?

[if required, their goals were: ____]

Part E. What works? Testing causal hypotheses

15. We have some ideas about what makes these yoga classes work for most people, but not everyone. Can I tell you those ideas and get your views on them? OK, so we think:

a. People anticipate *health benefits* from doing yoga and then they experience actual benefits. Was this true for you? What do you think makes people stick with classes early on, before any benefits can be felt? Why did you?

b. The *quality of yoga instructors* is important for making *people feel safe and confident about what they can achieve,* and this involves *taking account of people's different needs.* What do you think? How were your needs and preferences catered for?

c. *Social connections* are an incentive for some people and may add to the *enjoyment* of classes and even give *a sense of group belonging*. Was that your experience?

d. *Accessibility* is probably important and we're thinking about this in terms of costs, access to the studio for F2F classes and technological access for online classes. [and language for interviewees with NESBs]. *Convenience* might be part of that. What was your experience of accessibility?

e. Regular classes, twice a week over a year seem to have helped some people really *immerse themselves in yoga* and *build a routine* around it. Do you think that structure affected your engagement?

f. A few people have talked about *feeling challenged* by yoga in a good way, and their *desire to progress* and *feel they've achieved something*. Do you relate to that? [How] does this fit in with your goals?

g. Some people feel there's something different about yoga compared to other forms of exercise or recreation. Do you feel there's any difference? [PROMPTS: Maybe it's about *connecting body and mind*, or *becoming more mindful*, or it's just *a different way of being in your body*? What do you think?]

PART F. Final thoughts

16. Is there anything else you can tell us that might help us to improve programs like this and to support older people to be as active as possible and avoid falls?

Thank you so much for helping us with our research. It's been really helpful to hear about your experience.

Questions for SAGE yoga instructors

Informal preamble because they are partners in the research. Mention:

- Potentially compromised anonymity: there are only three of them and they know each other which means they might recognise each other even in deidentified reporting.
- Audio recording – check it's OK
- Ask if they would like to see/contribute to draft of main evaluation paper.

1. Can you tell us about your approach to yoga classes in the SAGE program?
2. How do your SAGE classes differ from other yoga classes you run?
3. What do you think is different in your approach to standard fall prevention classes?
4. What has the experience of running online classes been like for you?
5. Have you modified your teaching techniques for an online format?
6. What feedback have you had from SAGE participants about taking part in online classes?
7. What do you think is working for those who have stuck with classes?
8. What's your best guess about what wasn't working for those who dropped out?
9. We have several tentative theories about what works for the people who are sticking with SAGE. Can you please tell us what you think of them?
    a) People *anticipate health benefits* from doing yoga and then they *experience actual benefits*. If this is true, what makes people stick with classes early on, before any benefits can be felt?
    b) The *quality of yoga instructors* is important for making *people feel safe and confident about what they can achieve, and this involves taking account of people's different needs.*
    c) *Social interaction* is an incentive for some people and may add to the *enjoyment* of classes and even give a sense of *group belonging*.
    d) *Accessibility* is probably vital and we're thinking about this in terms of costs, access to the studio for F2F classes and then technological access for online classes. *Convenience* might be part of that.
    e) The frequency, duration and flexibility of classes creates *momentum* and encourages participants to *embed yoga in their daily routine*. Homework is probably part of this?
    f) *Yoga has special properties* that make it more satisfying than other forms of exercise. We think part of this is to do with concepts such as body/mind/spirit connection, embodiment and mindfulness. Do you intentionally facilitate this? How?
    g) We're also interested in the concept of *challenge and achievement* because this seems important in the data so far, but we haven't figured it out yet. Any thoughts?
10. What else should we know that will help us understand (a) how you are achieving such positive results with most SAGE participants, and (b) why it hasn't suited some participants?

Thank you!
